# Case Report: Fournier's gangrene in children: a report of three cases from a pediatric center in Vietnam

**DOI:** 10.3389/fped.2025.1728089

**Published:** 2026-01-21

**Authors:** Hung Thanh Le, Dao Thi Anh Nguyen, Trong Le Duc Vo, Linh Thi Truc Nguyen, Chan Cong Huynh, Phu Dai Tran, Cuong Trung Ho, Quynh Tran Minh Nguyen, An Tai Nguyen

**Affiliations:** 1 Department of Urology, Children’s Hospital, Ho Chi Minh City, Vietnam; 2 Department of Pediatric Surgery, University of Medicine and Pharmacy at Ho Chi Minh City, Ho Chi Minh City, Vietnam

**Keywords:** broad-spectrum antibiotics (ABX), debridement, fournier's gangrene, necrotizing fasciitis, pediatric

## Abstract

Fournier's gangrene (FG) is a form of necrotizing fasciitis affecting the perineal and external genital regions. It is a rare urological emergency, particularly uncommon in children. Accurate diagnosis, early administration of broad-spectrum antibiotics, and timely surgical debridement are essential for effective management. In this study, we present three distinct pediatric cases of FG. The first case involved a 10-month-old boy with no underlying health conditions who was successfully treated with favorable outcomes. The second case was a 12-year-old boy with dengue fever and extensive scrotal gangrene, who recovered well after a challenging postoperative course. The third case was a 4-year-old boy who developed necrotizing scrotal infection following a second-stage hypospadias repair. All three patients were promptly diagnosed and managed with early intervention.

## Introduction

Fournier's gangrene (FG) is a rare and rapidly progressing form of necrotizing fasciitis that affects both superficial and deep tissues of the perineum and external genitalia (1). FG constitutes a urological emergency and is particularly rare in pediatric populations. Global reports estimate its incidence at only 0.8 cases per 1 million individuals, with approximately 66% of pediatric FG cases occurring in infants under 3 months of age (2). First comprehensively described and named by Jean-Alfred Fournier in 1883, the condition was initially noted in five previously healthy men who developed sudden-onset, fulminant scrotal gangrene without identifiable cause (3). However, more recent studies have shown that FG in children is commonly associated with localized infections of the perineal fascia, scrotum, or external genitalia, and it can affect both males and females. In adults, FG typically occurs in the context of systemic immunosuppression such as diabetes mellitus, chronic alcoholism, malignancies, or HIV infection. In pediatric patients, while cases related to underlying malignancy have been documented, the majority occur in previously healthy children. Common predisposing factors include trauma (e.g., insect bites, circumcision, paraphimosis, incarcerated inguinal hernia), localized or systemic infections (e.g., diaper rash, varicella, perirectal infections), structural anatomical anomalies (e.g., urinary fistulas). The source of infection in FG cases is reported to originate from the external genitalia in 45% of cases, the anorectal region in 33%, and cutaneous sources in 21% (4). The use of single-stage debridement and primary skin closure in all three cases is noteworthy, as most pediatric series report staged reconstruction or diversion procedures (4–7). We report three cases of FG in children, two of which occurred at an atypical age (>3 months) and the cases are presented in ascending order of age.

## Case presentation

### Case 1

**Figure 1:**
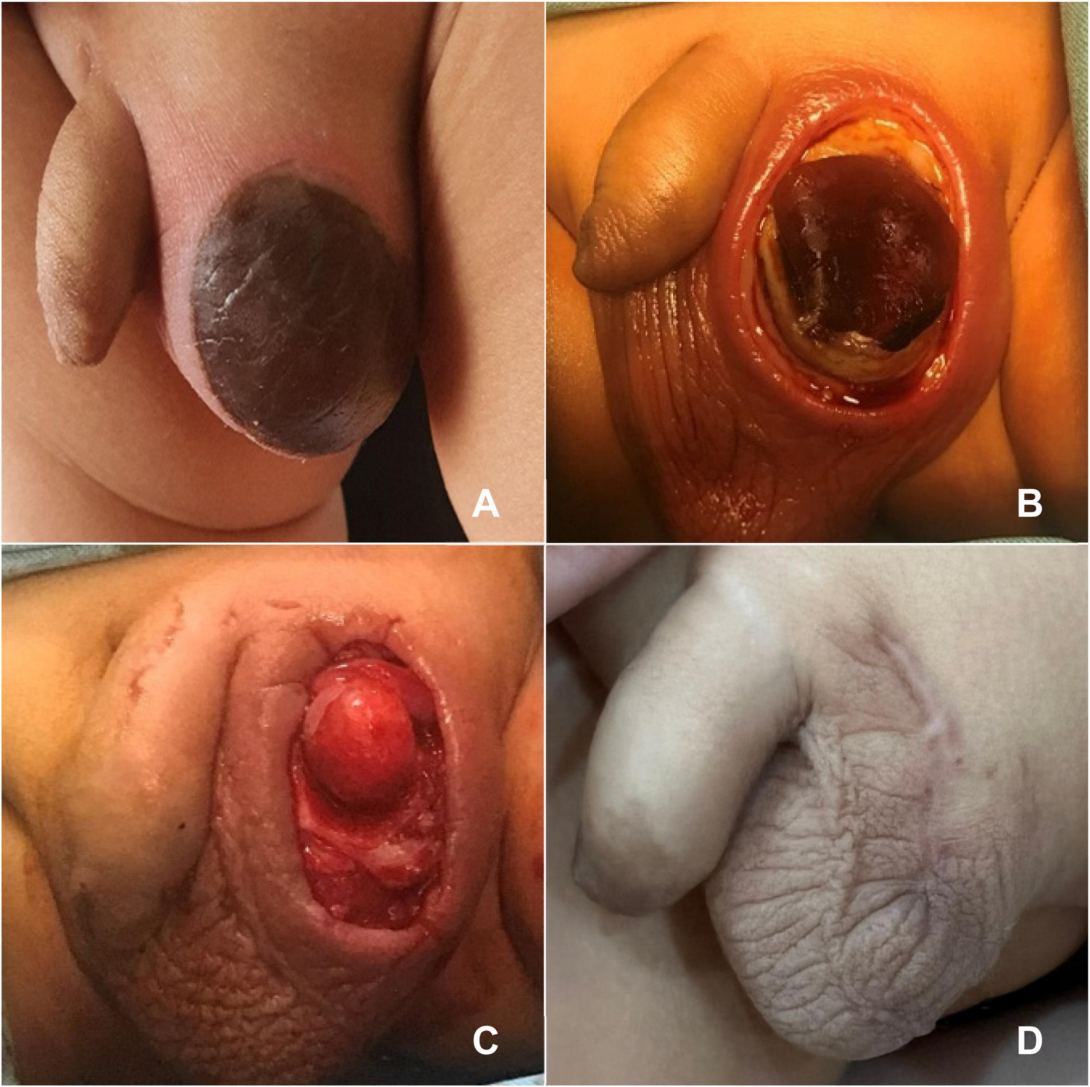
Appearance of the scrotal skin before and after debridement. **(A)** Admission Day; **(B)** Pre-debridement; **(C)** Post-debridement; **(D)** Complete Wound Healing.

A 10-month-old male presented with progressive scrotal swelling and pain over the course of seven days. Physical examination revealed an enlarging oval-shaped area of skin discoloration on the scrotum, tender to palpation (Figure 1A). The patient remained afebrile throughout the illness, with poor feeding but normal urination and defecation. Perinatal history was unremarkable, first child, full-term birth, with a birth weight of 3.2 kg and no known medical conditions.

On examination, the left scrotum was edematous with a sharply demarcated oval-shaped area of necrotic skin, dark purple-black in color, measuring approximately 4 × 2 cm. Both testes were present within the scrotum. Scrotal ultrasound showed homogenous echogenicity of both testes with preserved vascularity. A heterogeneous mass was detected in the left inguinoscrotal region, measuring 27 × 12 mm, with increased vascularity, fluid collection containing internal echoes, and gas formation.

Laboratory investigations showed a white blood cell count of 15,000/µL and C-reactive protein (CRP) of 4.73 mg/L. Gram-stain of the wound discharge revealed abundant polymorphonuclear leukocytes (+++), and Gram-negative bacilli (++). Wound cultures identified *Enterobacter cloacae* and *Escherichia coli* (non-ESBL producing).

Treatment from day 1 to day 5 included third-generation cephalosporins and metronidazole. On day 5, the patient underwent surgical debridement of necrotic tissue (Figures 1B,C). From day 5 to day 12, he continued to receive antibiotics and daily wound care. The wound healed well, and the patient was discharged on day 12 (Figure 1D).

### Case 2

A 4-year-old male with no prior medical history underwent a second-stage hypospadias repair. On postoperative (PO) day 1, he developed a high-grade fever of 40°C and was treated with antipyretics. On PO day 2, the fever persisted at 40°C without response to antipyretics. Clinical examination revealed scrotal swelling and erythema. Scrotal ultrasound demonstrated subcutaneous fluid collection. The patient was diagnosed with a surgical site infection and started on third-generation cephalosporins.

**Figure 2:**
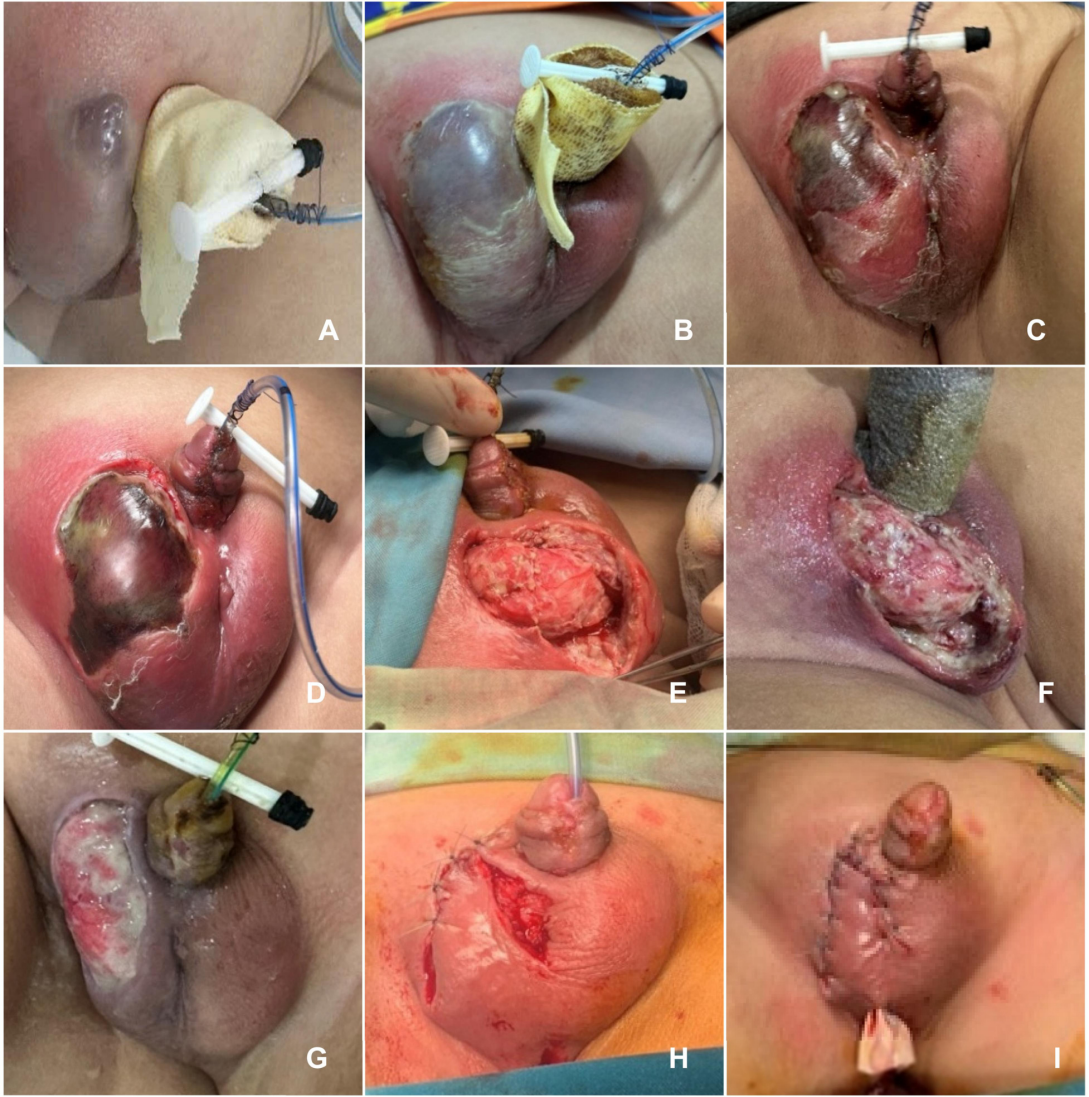
Development of necrosis lesion in a child after hypospadias repair. **(A)** Scrotal swelling and erythema on PO day 4; **(B,C)** On day 5, necrosis lesion appeared on the scrotum and spread rapidly; **(D,E)** Pre and Post-surgical debridement of necrotic tissue on PO day 6; **(F)** Post-surgical debridement of necrotic tissue on day 7; **(G)** The wound was gradually improving on day 13; **(H,I)** Secondary skin closure was performed with penrose drainage. PO, postoperative.

On PO days 3–4, the patient remained febrile, prompting the addition of gentamicin and metronidazole (Figure 2A). On day 5, bullae appeared on the scrotum with rapid extension of tissue damage (Figures 2B,C). Fournier's gangrene of the scrotum, post-hypospadias surgery, was suspected. Laboratory results showed a white blood cell count of 27,000/µL and CRP level of 182 mg/L. Cephalosporins were discontinued, and treatment was escalated to vancomycin and imipenem.

By 9:00 PM the same day, the patient remained febrile, with widespread foul-smelling scrotal necrosis. Incision and drainage were performed, and a surgical drain was placed. On PO day 6, surgical debridement of necrotic tissue was carried out (Figures 2D,E), and the antibiotic regimen was adjusted to include meropenem, vancomycin, metronidazole, and gentamicin. A blood sample was also sent for multiplex PCR testing.

On PO day 7, wound cultures identified multidrug-resistant Escherichia coli, sensitive to imipenem and meropenem (Figure 2F). The patient's clinical status gradually improved (Figure 2G). From days 7 to 14, local wound care was administered using Granudacyn® solution and Mepilex Ag dressings. On PO day 14, secondary skin closure was achieved using a rotational skin flap to cover the defect (Figures 2H,I).

### Case 3

A 12-year-old male was initially diagnosed with dengue fever and managed as an outpatient for three days. He was subsequently admitted to the hospital due to progressively worsening scrotal swelling and pain, accompanied by the appearance of multiple necrotic skin patches. On examination, the scrotum was tender and edematous, with necrotic skin lesions observed at the base of the scrotum.

Scrotal ultrasound revealed scattered hemorrhage within the scrotal tissue; both testes appeared homogeneous and preserved. Laboratory investigations showed a white blood cell count of 12,000/µL and CRP level of 4 mg/L. Gram staining of wound discharge showed abundant polymorphonuclear leukocytes (+++) and Gram-negative bacilli (++). Wound cultures yielded *Pseudomonas aeruginosa*, *Klebsiella pneumoniae* (ESBL-positive), and *Citrobacter freundii*.

**Figure 3:**
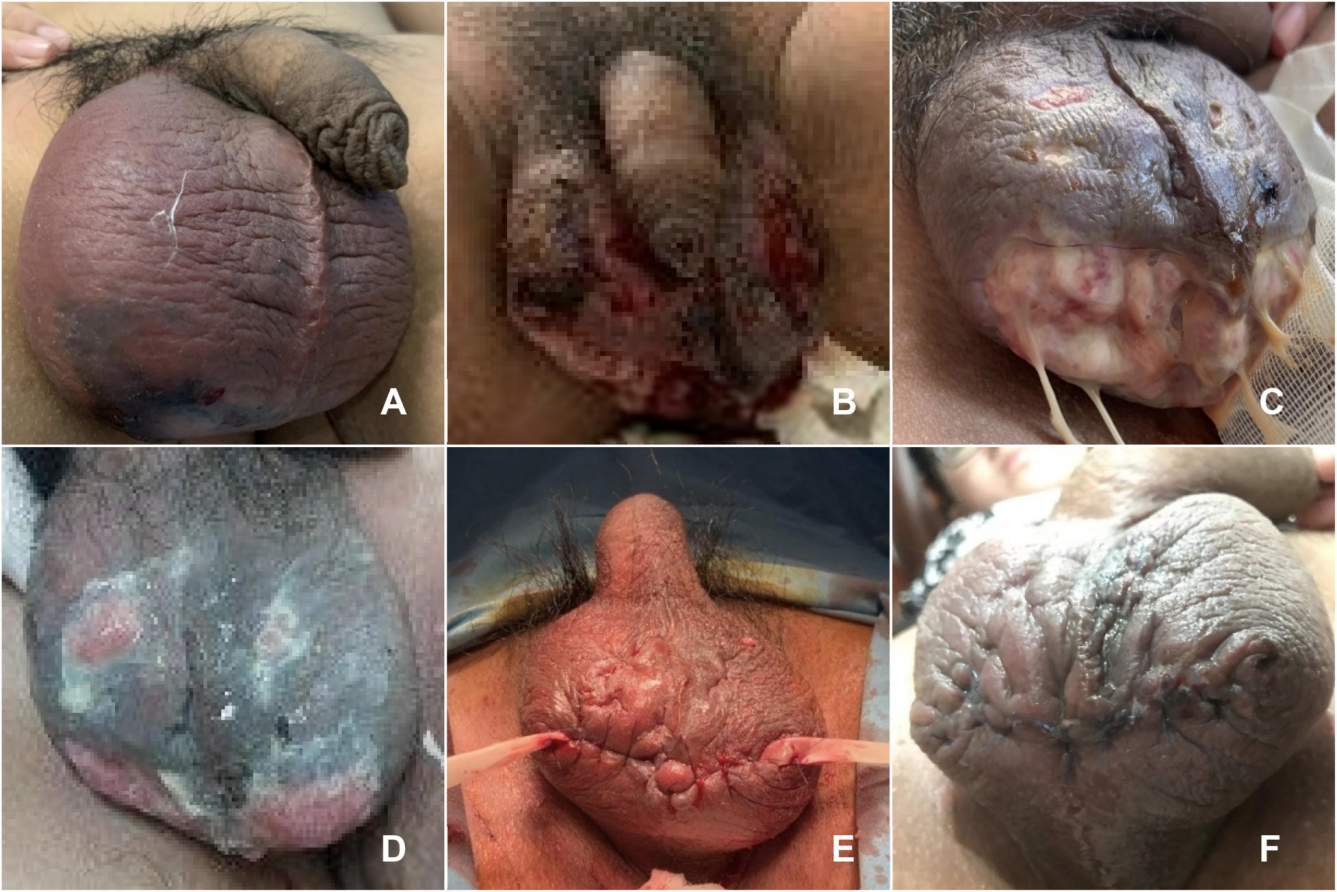
Bilateral scrotal Fournier's gangrene and postoperative wound healing. **(A)** Srotal swelling on day 5; **(B)** Progressive necrosis of the scrotal skin on day 7; **(C)** Full-thickness scrotal skin necrosis with exposed subcutaneous tissue on day 10; **(D)** The scrotal skin was gradually healing, with granulation tissue established by day 19; **(E)** Post-surgical debridement with penrose drain placement on day 20; **(F)** The wound healed satisfactorily on day 24.

From days 1 to 10, the patient was treated with third-generation cephalosporins and metronidazole. From day 10 to 19, the necrosis progressed with extensive skin and subcutaneous tissue loss and yellow purulent discharge (Figures 3A,C). The antibiotic regimen was switched to vancomycin and metronidazole, along with local wound care using Granudacyn® and hydrogen peroxide 2–3 times daily. By day 19, the scrotum showed reduced purulence and development of thick granulation tissue (Figure 3D).

On day 20, the patient underwent surgical debridement with penrose drain placement (Figure 3E). From day 20 to 24, antibiotic therapy and wound care were continued. The wound healed satisfactorily, and the patient was discharged on day 24 (Figure 3F).

## Discussion

FG is a rapidly progressing form of necrotizing fasciitis that affects both superficial and deep tissues of the perineum, anorectal region, and scrotum, typically caused by polymicrobial infections. The causative organisms produce enzymes such as collagenase and hyaluronidase, which facilitate the rapid spread of infection along fascial planes, leading to vascular thrombosis and subsequent necrosis of overlying skin (5). The pathogens are usually a mix of aerobic bacteria (e.g., *Streptococcus spp.*, *Staphylococcus aureus*, *Escherichia coli*, *Pseudomonas aeruginosa*) and anaerobic bacteria (e.g., *Clostridium spp.*, *Bacteroides spp.*). Wound cultures typically yield multiple organisms, with an average of four different species per sample (5, 8). Similar findings were reported in studies by Mohsen Rouzrokh (6) (7 cases) and Singh Bains (5) (1 case), whose wound cultures also revealed a polymicrobial profile comparable to ours.

The anatomical continuity between Colles’ fascia of the genital-perineal region and Dartos fascia of the lower abdomen, both supplied by branches of the inferior epigastric artery and pudendal arteries, facilitates the rapid spread of infection. However, the testicular blood supply originates directly from the abdominal aorta, which likely explains why testicular viability was preserved in all three of our cases (5).

According to the literature, 66% of pediatric FG cases occur in infants under 3 months of age, making the age group in our series atypical. Although FG was initially described as idiopathic, approximately 95% of cases are now known to originate from infections in the anorectal region, urinary tract, or perineal skin. Reported pediatric risk factors include incarcerated hernias, prematurity, diaper rash, varicella, circumcision, perineal abscesses, and nephrotic syndrome. Additional predisposing factors include invasive procedures involving the perineum or urethra, burns, and systemic infections (5). Among our cases, one was associated with dengue fever, another occurred following hypospadias surgery and one had no identifiable trigger. To the best of our knowledge, there are currently no reports linking Dengue fever to FG. However, studies on peripheral gangrene suggest that Dengue fever increases vascular permeability with coagulation disorders, thereby potentially causing occlusion of the small vessels in the perineum in FG (9).

The diagnosis of FG is primarily clinical, based on signs such as pruritus, erythema, pain, swelling, and skin discoloration in the perineal, scrotal, or labial regions (10). In early stages, it may be mistaken for less severe dermatologic or infectious conditions. As the disease progresses, skin changes to a purple or black hue, with bullae formation and foul odor indicative of anaerobic bacterial activity. Crepitus may be noted on palpation (1, 10). FG scoring systems (e.g., FGSI) were not applied in our report as they have limited validation in pediatric populations. Laboratory findings typically include leukocytosis and elevated CRP. Imaging such as plain radiographs may show subcutaneous gas in up to 90% of cases. Ultrasound is useful for detecting gas and evaluating testicular perfusion (1). All three of our cases followed a clinical and diagnostic trajectory consistent with these findings. None of our patients underwent CT scans or radiographs because ultrasound already demonstrated subcutaneous gas, fluid collections, and preserved testicular perfusion, which were adequate for diagnosis and decision-making.

Although earlier studies reported mortality rates as high as 40%–45% (5). Mohsen Rouzrokh's series of seven pediatric cases (ages 6 months to 8 years) reported a 42.86% mortality rate (3/7) due to septic shock and multiorgan failure despite appropriate management (6). Mortality remains significant but has decreased with modern multidisciplinary management. Contemporary reviews describe mortality as variable but generally lower than historical estimates, reflecting improved outcomes in both adult and pediatric populations (11). None of our patients developed sepsis or multiorgan dysfunction, likely due to early diagnosis and prompt intervention. As a result, all three cases had favorable outcomes (survival, discharge, complete wound healing).

The cornerstone of FG management is a combination of broad-spectrum antibiotics and prompt surgical debridement. Delays in surgical intervention are associated with increased mortality (1, 2).. In our series, the timing of surgical debridement was individualized based on clinical progression rather than performed immediately at presentation. Debridement was undertaken once patients demonstrated hemodynamic stability, clearer demarcation of necrotic tissue, and partial response to early broad-spectrum antibiotics. This approach was chosen to avoid excessive removal of viable tissue and to minimize the need for multiple procedures. In all three cases, surgical timing was guided by daily clinical assessment, wound appearance, inflammatory marker trends, and imaging findings. This strategy contributed to stable postoperative recovery and allowed for successful single-stage closure in each patient. All three cases underwent single-stage debridement and primary skin closure. This differs from reports by Mohsen Rouzrokh (6), Singh Bains (5), Cundy (4) and Ekingen (7), where staged closures or diversion procedures (e.g., colostomy, vesicostomy) were required to prevent fecal or urinary contamination. This variation may be attributed to early diagnosis and antibiotic treatment in our series, and the absence of septic or organ failure states. Several authors have reported the use of adjunctive measures such as vacuum-assisted closure (VAC) therapy to optimize wound healing in extensive perineal defects. Recent case-based evidence supports VAC as a useful adjunct following debridement, particularly in cases with large soft-tissue loss (10, 12).

Wound care played a critical role in our management. We utilized a combination of antimicrobial solutions including hypochlorous acid (Granudacyn®) and hydrogen peroxide (H₂O₂), similar to protocols described by Rouzrokh (0.5% H₂O₂ with 1% citric acid) and Cundy (1.5% H₂O₂ with povidone-iodine) (4).

## Conclusion

Fournier's gangrene is a rare urological emergency in pediatric patients. Early diagnosis, timely administration of broad-spectrum antibiotics, combined with surgical debridement and appropriate wound care, plays a critical role in reducing mortality and minimizing complications.

## Data Availability

The original contributions presented in the study are included in the article/Supplementary Material, further inquiries can be directed to the corresponding author.
